# Lateral inhibition of Notch signaling in neoplastic cells

**DOI:** 10.18632/oncotarget.2762

**Published:** 2014-12-09

**Authors:** Kah Jing Lim, William D. Brandt, Jason A. Heth, Karin M. Muraszko, Xing Fan, Eli E. Bar, Charles G. Eberhart

**Affiliations:** ^1^ Department of Pathology, Johns Hopkins University, Baltimore, MD 21231, Maryland, USA; ^2^ Department of Oncology, Johns Hopkins University, Baltimore, MD 21231, Maryland, USA; ^3^ Department of Ophthalmology, Johns Hopkins University, Baltimore, MD 21231, Maryland, USA; ^4^ Department of Neurosurgery, University of Michigan, Ann Arbor, MI 48109, Michigan, USA; ^5^ Department of Cell and Developmental Biology, University of Michigan, Ann Arbor, MI 48109, Michigan, USA; ^6^ Department of Neurological Surgery, Case Western University, Cleveland, OH 44106, Ohio, USA

**Keywords:** Notch, lateral inhibition, cancer, signaling

## Abstract

During normal development, heterogeneous expression of Notch ligands can result in pathway suppression in the signal-sending cell, a process known as lateral inhibition. It is unclear if an analogous phenomenon occurs in malignant cells. We observed significant induction of Notch ligands in glioblastoma neurospheres and pancreatic carcinoma cells cultured in low oxygen, suggesting that this phenomenon could occur around hypoxic regions. To model lateral inhibition in these tumors, the ligand Jagged1 was overexpressed in glioblastoma and pancreatic carcinoma cells, resulting in overall induction of pathway targets. However, when ligand high and ligand low cells from a single line were co-cultured and then separated, we noted suppression of Notch pathway targets in the former and induction in the latter, suggesting that neoplastic lateral inhibition can occur. We also found that repression of Notch pathway targets in signal-sending cells may occur through the activity of a Notch ligand intracellular domain, which translocates into the nucleus. Understanding how this neoplastic lateral inhibition process functions in cancer cells may be important in targeting ligand driven Notch signaling in solid tumors.

The Notch pathway is active and functionally important in a wide range of tumors, including hematopoietic neoplasms [1, 2], melanoma [3], and carcinomas of the breast [4, 5], ovary [6], lung [7], prostate [8], and pancreas [9]. In many of these tumors, Notch is believed to play key roles in the specification, proliferation and survival of treatment-resistant cancer stem cells [10–12]. Understanding how Notch signaling is initiated and maintained is therefore critical for effective therapeutic intervention in a range of tumor types.

In non-neoplastic contexts, Notch activity is initiated when ligands from the Jagged (JAG) and Delta-like families on one cell bind to Notch receptors on an adjacent cell. The receptors on the cell surface are processed through two sequential cleavages. First, the ADAM family of secretases cleaves the receptor to produce the Notch C-terminal fraction. Next, γ-secretase cleavage within the membrane leads to release of the Notch intracellular domain (NICD), which translocates into the nucleus of the signal-receiving cell. NICD then binds co-factors, including CSL, MAML and other co-activators, to induce transcription of target genes in the *HES* and *HEY* families.

In a subset of cancers, including T cell ALL [13], breast [14], and lung cancer [15], Notch is activated by mutations or translocations that directly alter receptors or other key pathway members (reviewed in: [16, 17]). In most tumors, however, Notch signaling is initiated when receptors on the tumor bind to ligands expressed by adjacent cells. In some tumor microenvironments, Notch ligands are highly expressed on blood vessels [18, 19], inflammatory cells [20–22] or other stromal elements [23–25], thus signaling is from non-neoplastic cells to cancerous ones. In other contexts, however, tumor cells themselves are known to express both ligands and receptors, and it is thought that signaling between neoplastic cells is a major driver of Notch activity [26, 27].

During normal development, several mechanisms are used to regulate Notch activity when groups of similar cells express both ligand and receptor, with the best studied of these being lateral inhibition. This process, first described in *Drosophila*, allows the specification of evenly spaced neuroblasts from sheets of undifferentiated epithelial progenitors via feedback loops that reinforce initial differences in ligand and receptor levels between adjacent cells [28, 29]. In the signal-sending cell, generation of a JAG intracellular domain protein [30] is believed to actively repress Notch signaling either by directly binding and sequestering NICDs [31, 32] or by binding the Notch transcriptional machinery in the nucleus [33]. While this lateral inhibition paradigm is commonly accepted in developmental biology, to our knowledge it has not been well studied in solid tumors expressing both ligands and receptors on neoplastic cells. We therefore decided to address the possibility that a lateral inhibition-like process occurs in cancer, using the brain tumor glioblastoma (GBM) as our primary model.

GBM is the most common primary malignant brain tumor. The median survival of patients with this devastating disease is still approximately one year, even after surgical resection, radiation and chemotherapy with temozolomide [34, 35]. The Notch signaling pathway is increasingly being identified as an important player in the growth and survival of GBM [27, 36, 37], including cancer stem cells in these tumors [38, 39]. This small population of stem-like GBM cells supported by Notch activity appear to be associated with resistance to standard radiation and chemotherapy [38, 40–42].

Notch has been shown to be active in the GBM vascular stem cell niche [39], with signaling driven by Notch ligands present on endothelial cells [19]. However, it has also been demonstrated that Notch activity is upregulated in glioma cells grown in low oxygen culture [43], and in regions of hypoxia *in vivo* [43–45]. This suggests that at least two alternate microenvironments supporting Notch activity may exist in GBM, a perivascular niche with ligands expressed on vascular elements, and a peri-hypoxic niche with ligands induced on tumor cells. The latter environment, in which both ligands and receptors are expressed on adjacent or intermixed tumor cells, might represent a region in which asymmetry in expression leads to lateral inhibition.

In this study, we examine in greater detail the effects of hypoxia on Notch ligand expression in GBM and pancreatic carcinoma. We also sought to directly model what happens when adjacent tumor cells express differing levels of ligand, identifying a lateral inhibition-like phenomenon. We also reviewed images from three separate primary GBM specimens from a prior study in which we stained for both Notch ligands and targets [19]. In all three samples, regions of adjacent neoplastic cells expressing either ligand or target were readily identified, supporting the possibility of lateral inhibition *in vivo*. Finally, we show that ligand ICD is able to mediate effects similar to those seen in lateral inhibition.

## RESULTS

### Hypoxia induces expression of the notch ligands JAG1 and JAG2 in GBM neurospheres

**Figure 1:**
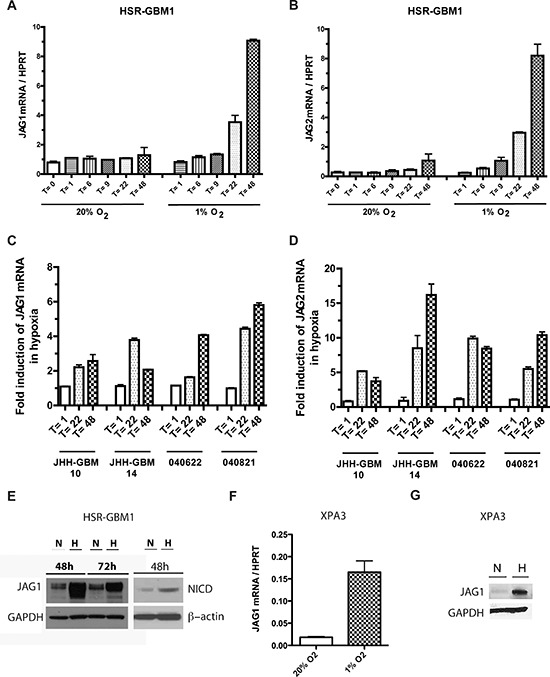
Hypoxia induces Notch ligand expression in malignant tumors HSR-GBM1 neurospheres were subjected to normoxia (20% O_2_) or hypoxia (1% O_2_) for 1 (*T* = 1), 6 (*T* = 6), 9 (*T* = 9), 22 (*T* = 22) and 48 (*T* = 48) h, at which time they were collected for RNA analyses. **(A)** JAG1 and **(B)** JAG2 mRNA levels were both induced soon after hypoxia exposure. **(C)** JAG1 and **(D)** JAG2 mRNA levels were similarly upregulated in the GBM neurospheres lines JHH-GBM10, JHH-GBM14, 040621 and 040821. **(E)** JAG1 and NICD protein levels were also induced following 48 h exposure to hypoxia, with JAG1 expression enduring at 72 h. The pancreatic cancer cell line XPA3 also induces JAG1 **(F)** mRNA and **(G)** protein levels following 48 h exposure to hypoxia.

Previous work from our laboratory demonstrated that hypoxia can positively regulate mRNA levels of the Notch ligands JAG1 and JAG2 in GBM neurospheres [43]. We investigated this further in a larger panel of GBM neurosphere lines in which we also assessed the effects of neurosphere growth on ligand levels and pathway activity over several days. Following hypoxia induction in HSR-GBM1 neurospheres, we observed the upregulation of JAG1 and JAG2 ligands beginning at 22 hours and 9 hours, respectively (Figure 1A, 1B). We also observed a several-fold increase in mRNA levels of the Notch ligands JAG1 and JAG2 at both 22 and 48 hours post hypoxia induction in the GBM lines JHH-GBM10, JHH-GBM14, 040622 and 040821 (Figure 1C, 1D).

We then extended this analysis to protein, and found levels of ligand induction similar to those seen at the mRNA level. In the HSR-GBM1 line, for example, JAG1 protein was upregulated over 10-fold as shown in Figure 1E. The cleaved (active) from of Notch1 (NICD1) was also induced, suggesting that the increases in ligand level may be driving pathway activity (Figure 1E). The pancreatic cancer cell line XPA3, which is known to be Notch-dependent [46], also showed dramatic upregulation of both JAG1 mRNA and protein in hypoxia (Figure 1F, 1G).

### Unequal JAG1 levels in co-cultured cells alters notch signaling in both the signal sending and receiving cells

**Figure 2:**
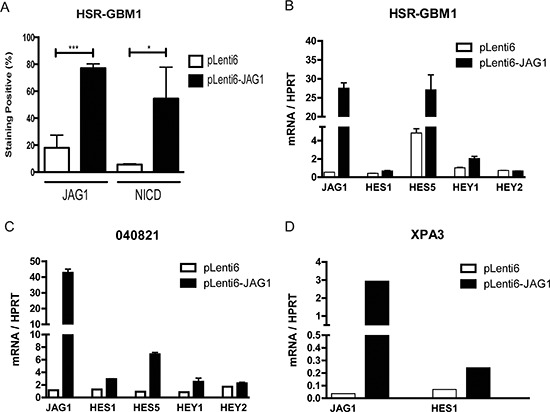
Ectopic ligand expression induces Notch signaling **(A)** Lentiviral transduction of *JAG1* resulted in 80% of cells expressing ligand compared to 20% in the control based on three separate immunofluorescence studies (Supplemental Figure 1). The percentage of cells expressing NICD was upregulated approximately 10-fold. Overexpression of JAG1 ligands in **(B)** HSR-GBM1, **(C)** 040821 and **(D)** XPA3 lines upregulates Notch targets from the HES and HEY families. **p < 0.01, ***p < 0.001 compared to pLenti6 control from 3 experiments.

To test the effects of increased Notch ligand levels, we generated GBM neurosphere lines that have elevated ligand levels. We induced JAG1 expression in the HSR-GBM1 and 040821 neurosphere lines via viral transduction followed by selection with blasticidin antibiotics. These bulk ligand-transduced cultures were found to increase the number of cells expressing JAG1 from approximately 20% to 80%, as measured by 3 separate immunofluorescent positive cell counts (Figure 2A, Supplemental Figure 1). However, even in the overexpressing cells, JAG1 protein levels varied significantly (Supplemental Figure 1).

This heterogeneous ligand induction resulted in increased levels of Notch pathway activity. The percentage of NICD expressing cells rose approximately 10-fold (Figure 2A, Supplemental Figure 1). Upregulation of the HES1, HES5 and HEY1 targets was also noted in both HSR-GBM1 (Figure 2B) and 040821 (Figure 2C) GBM neurospheres, but was generally less dramatic than the change in the percentage of NICD expressing cells. Overexpression of JAG1 also induced HES1, but not HEY1, in the pancreatic cancer cell line XPA3 (Figure 2D).

**Figure 3:**
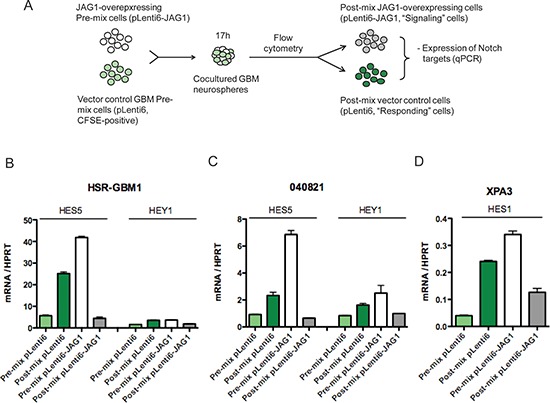
JAG-low cells are primed to respond to Notch receptor signals **(A)** In the co-culture experiments, GBM pLenti6 control cells were stained with CFSE and mixed with isogenic cells overexpressing JAG1 ligands (no CFSE stain) and co-cultured. After co-culture for 17 h, cells were sorted into their respective initial populations using the CFSE staining and analyzed by qPCR. **(B)** HES5 and HEY1 transcripts were upregulated following co-culture in pLenti6 (B) HSR-GBM1 and **(C)** 040821 cells while the same transcripts were downregulated in pLenti6-JAG1 cells. Similarly, following co-culture, HES1 transcripts were upregulated in **(D)** XPA3 pLenti6 cells but downregulated in pLenti6-JAG1 cells. **p < 0.01, ***p < 0.001 compared to pre-mix control.

To more directly model how unequal JAG1 levels in the hypoxic microenvironment, or other areas where they are not uniformly expressed, might affect Notch activity, we engineered a system in which JAG1 was elevated in a more controlled subset of cells. To achieve this, cells overexpressing JAG1 and vector transduced controls with lower levels of the ligand were co-cultured. These two populations were then sorted using flow cytometry and levels of pathway targets were measured by quantitative PCR (Figure 3A). The effects of co-culture on Notch activity were dramatically different in these two populations. Vector transduced (JAG1-low) cells in the two GBM cultures examined showed a significant induction of pathway targets when brought in contact with JAG1-high cells of the same line (light and dark green bars in Figure 3B, 3C). In contrast, the high baseline levels of Notch activity in cultures transduced with JAG1 were dramatically suppressed after co-culture with sister cells differing only in their lower ligand expression (white and grey bars in Figure 3B, 3C). We also observed similar effects on HES1 transcript levels following co-culture of JAG1-high and -low XPA3 pancreatic cancer cells (Figure 3D). Sorting either JAG1 high or low GBM1 cells without co-culture resulted in a modest elevation of HES5 expression, suggesting that the pathway inhibition on co-culture was not a side effect of flow cytometric manipulation (data not shown). These observations suggest that lateral inhibition, a mechanism specifying cell fate during development by downregulating pathway activity in ligand-high “signal sending” cells, can also occur in tumors.

### The JAG1 intracellular domain reduces notch signaling and decreases clonogenic growth in GBM neurospheres

**Figure 4:**
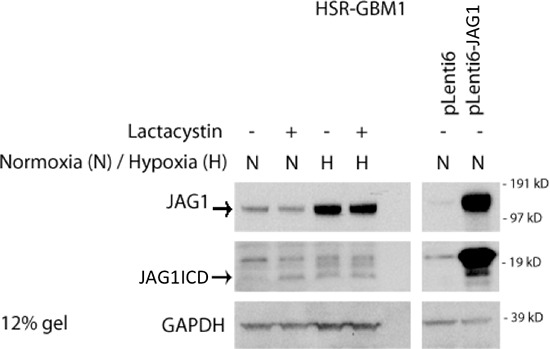
Hypoxia and JAG1 overexpression increases ligand ICD production Hypoxia induces full length JAG1 production and increases ICD (~16 kD) stability. Similarly, overexpression of JAG1 increases the expression of the ligand ICD band.

We next investigated the mechanism causing downregulation of Notch target gene levels in JAG1-high GBM cells following co-culture with paired JAG1-low cells. Like the Notch receptor, JAG ligands are also subjected to α-secretase and γ-secretase cleavage, which leads to the production of intracellular domain (ICD) proteins able to translocate into the nucleus and affect pathway activity [30, 33, 47–49]. We investigated ligand ICD levels in GBM neurospheres after hypoxia exposure or JAG1 overexpression. A low molecular weight band of a size consistent with ligand ICD appeared in hypoxia (Figure 4). It was absent under standard normoxic conditions, but was detectable in the presence of the proteasome inhibitor lactacystin. Similarly, overexpression of Jagged1 in GBM neurospheres led to an increase in ICD production (Figure 4). These results suggest that the cleaved ligand ICD may modulate Notch activity in GBM neurospheres.

**Figure 5:**
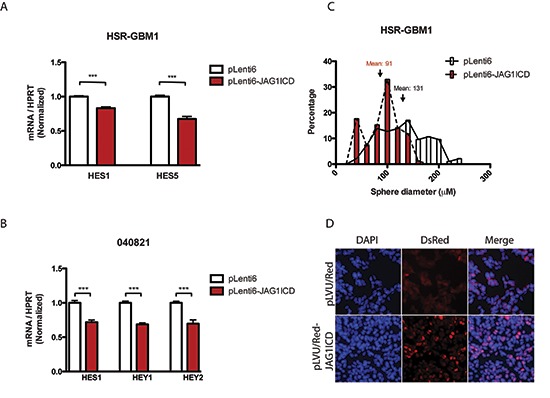
JAG1 ligand ICD reduces Notch signaling, potentially through nuclear localization Notch target levels were reduced in **(A)** HSR-GBM1 and **(B)** 040821 cells transduced with ligand ICD. The average sphere diameter of **(C)** HSR-GBM1 cells transduced with ligand ICDs was reduced in a clonogenic assay. **(D)** HSR-GBM1 cells transduced with C-terminus DsRed tagged JAG1ICD showed distinct nuclear localization, in distinct contrast to the pLVU/Red vector control transduced cells.

We therefore investigated the effects of overexpression of ligand ICD in our GBM cells. We first generated human JAG1 ligand ICD constructs and cloned them into viral vectors. GBM neurospheres were transduced with viruses containing the control vector pLenti6 or pLenti6-JAG1ICD and were subsequently selected with blasticidin antibiotics. We observed a modest but significant reduction in HES and HEY transcripts in ligand ICD overexpressing HSR-GBM1 and 040821 cells (Figure 5A, 5B), consistent with the concept that cleavage and release of the ligand ICD in the “signal sending” cell can suppress Notch pathway activity.

We have previously shown that the size of anchorage-independent colonies correlates with either their derivation from GBM cancer stem cells or more restricted progenitors, as colonies over 100 μM in size can be serially passaged, while those less than 50 μM cannot [43]. Thus, we next investigated the ability of GBM cells to form colonies over several weeks following ligand ICD overexpression. Approximately 20,000 HSR-GBM1 cells were seeded in methylcellulose and grown for 10–13 days. Subsequent sphere formation was monitored and scored by light microscopy. The mean diameter of cells infected with the control vector pLenti6 was 131 μM while cells infected with JAG1ICD were 93 μM (Figure 5C). There was a significant difference (*p* < 0.001) in the size of pLenti6 and pLenti6-JAG1ICD spheres, consistent with the concept that ligand ICD expression can suppress clonogenic capacity.

To further investigate the mechanism by which ligand ICD inhibits Notch signaling and reduces growth and clonogenicity in GBM cells, we examined its subcellular localization. After transducing HSR-GBM1 cells with pLVU/RED (C-terminal DsRed-tagged) JAG1ICD, fluorescent microscopy analysis showed that JAG1ICD localizes predominantly in the nucleus (Figure 5D), suggesting that like the Notch receptor ICD, ligand ICD may act in the nucleus to modulate transcription. This may explain why the induction of NICD we observed in Figure 2 was more prominent than that of transcriptional targets of the pathway.

## DISCUSSION

To most effectively target Notch in cancer, it will be necessary to understand in which subsets of cells it is active and how this activation is achieved. Notch ligands have been found to be upregulated in many types of cancer, and such ligands expressed on tumor cells themselves may provide the driving force behind pathway induction in some contexts. We therefore investigated the role of Notch ligands in GBM and pancreatic carcinoma, focusing on their induction in a hypoxic tumor microenvironment and the possibility that heterogeneous ligand levels might have complex effects on pathway induction analogous to those seen in normal development.

A recent study of JAG1 expression by immunohistochemistry in pseudopalisading GBM tumor cells suggests that hypoxia can focally promote its expression in tumor cells surrounding necrotic regions, resulting in unequal ligand levels [44]. We confirmed the dramatic induction of Notch ligand mRNA and protein in GBM and pancreatic carcinoma cultures when placed in hypoxic conditions (Figure 1). However, our data also suggest that unequal JAG1 ligand levels in a mixed tumor cell population results in dramatically different effects on the heterogeneous cells.

**Figure 6:**
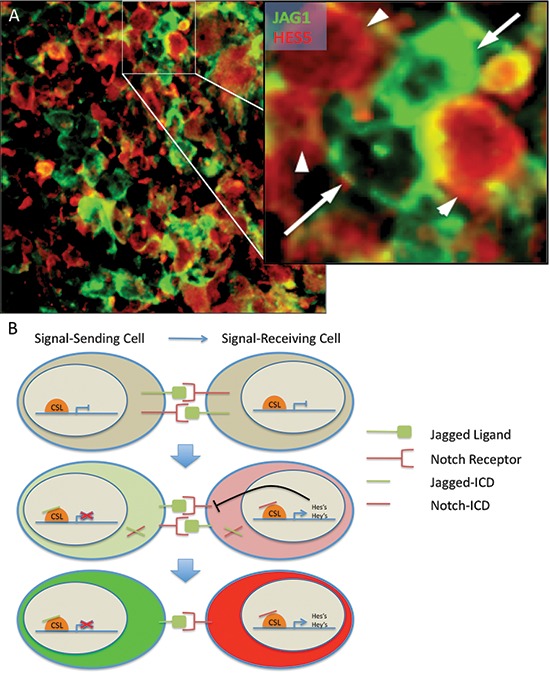
Modeling Notch signaling in GBM **(A)** Notch regulates differentiation through lateral inhibition, whereby activation of the pathway results in the production of a ligand ICD as well as a receptor ICD. The ligand ICD inhibits NICD-driven transcription in the signal-sending cell whereas NICD induces transcription in the signal-receiving cell. **(B)** Expression of JAG1 and HES5 are generally mutually exclusive. Notch ligand JAG1-expressing cells (green, arrow) are adjacent to, but mostly mutually exclusive from, Notch target HES5-expressing cells (red, arrowhead) in a primary GBM tumor sample. Similar results were seen in the two additional cases analyzed.

In many ligand-receptor pairs, increased ligand expression leads to increased signaling in the receiving cell, but no decrement in the sending cell. For the Notch pathway, lateral inhibition occurs when differential expression of ligands in adjacent cells leads not only to induction of Notch signaling in the ligand-low cell, but also to pathway inhibition in the ligand-high cell (Figure 6A). In normal development, this results in the specification of distinct cell types from an undifferentiated field [50].

Our study suggests that an analogous process can occur in malignant cells. Two tumor systems were examined: GBM neurospheres and an adherent pancreatic carcinoma line. In both tumor types, introduction of JAG1 into the cancer cells increased overall Notch signaling levels in the culture, but we still observed heterogeneity in ligand levels, which likely facilitated this overall pathway induction and made it difficult to separate signal sending and receiving cells. However, when ligand high cells were co-cultured with the ligand low matched controls, the changes in Notch signaling recapitulated those seen in classical lateral inhibition. Following co-culture and sorting, pathway targets were increased in ligand low (signal-receiving) cells, while the same targets were greatly reduced from baseline in high JAG1 (signal-sending) cells.

A number of prior reports have indicated that Notch receptors, ligands and/or targets are heterogeneously expressed in primary glioma [27, 44, 51–53] and pancreatic carcinoma [54–56] specimens, suggesting that a lateral inhibition like phenomenon could occur in patients. We investigated if the mutually exclusive expression of a Notch pathway ligand (JAG1) and target (HES5) consistent with lateral inhibition could be found in primary glioblastoma by examining in greater detail three previously reported, double stained GBM specimens [19]. In all three cases examined, while the relative proportions of ligand and target expressing cells varied significantly, they were in general roughly equivalent in number, with a much smaller population of cells that appeared to express both ligand and target. Tightly stereotyped patterns of expression similar to those described in developing *Drosophila* epithelium were not observed. However, we estimate that 40–60% of the overall tumor areas examined showed a largely mutually exclusive pattern of expression in neoplastic-appearing cells, as illustrated in Figure 6B. While larger numbers of cases and additional ligands and targets will need to be examined to determine the full prevalence and impact of lateral inhibition in tumors of the brain and other tissues, our preliminary studies support the general notion that such a process can occur *in vitro* and *in vivo*.

To explore the mechanism by which Notch signaling is reduced in JAG1-high cells, we looked at protein expression under hypoxia and in the context of ligand overexpression. Both conditions resulted in increased JAG1 ICD protein predominantly localized to the nucleus of the cell. Overexpression of ligand ICD was found to reduce Notch target expression as well as clonogenicity of GBM neurospheres. Recent studies have demonstrated nuclear localization of Notch ligands in *Drosophila* [47], mouse [30] and rat [33]. In addition, potential nuclear localization signals (NLS) have previously been identified in human *DLL1* [57]. It is therefore possible that the attenuation of Notch activity could be linked to the nuclear localization of ligand ICDs. Our results also support previous reports by Jung et al and Kim et al, which suggested that the ligand ICD can attenuate Notch signaling via translocation into the nucleus and binding to NICD [31, 32].

To our knowledge, this is the first specific investigation of lateral inhibition occurring in tumor cells. A recent study on adrenocortical carcinoma used a similar co-culture strategy to demonstrate that JAG1 functions in a non-cell-autonomous manner to induce signaling, and is at least partially responsible for aggressive cellular proliferation [58]; however, pathway suppression in the ligand high cells was not examined. Understanding the heterogeneity of Notch activity in tumors, how they relate to microenvironmental factors such as hypoxia, and the sources of ligand that drive the pathway may allow us to more rationally target Notch in cancer. The finding that JAG1 ligand ICD can suppress pathway activity and tumor growth also represents a new possible therapeutic strategy.

## MATERIALS AND METHODS

### Immunofluorescence

Primary GBM tumor samples were fixed in 4% paraformaldehyde overnight at 4°C, cryoprotected with 30% sucrose saturation, and cryoembedded in Tissue-Tek OCT. Tissue sections (8 μm thick) were permeabilized with 0.4% Triton-X-100 for 25 min at room temperature and blocked with 5% BSA in PBS for 20 min at room temperature. Samples were incubated with primary antibodies overnight at 4°C and secondary antibodies were applied for 1 h at room temperature. Primary antibodies and dilutions used for immunofluorescence were JAG1 (Cell Signaling, 2155, 1:100), HES5 (Santa Cruz, sc-13859, 1:50), and cleaved-NOTCH1 (Cell Signaling, 4147, 1:100). Secondary antibodies used were Cy2 or Cy3 conjugated antibody (1:500, Jackson Immuno Research). For cleaved-NOTCH1 immunofluorescence, and the JAG1 immunofluorescence in Figure 2A and Supplemental Figure 1, a Powervision poly-HRP-anti-rabbit secondary (Dako) was used, followed by Cy3 TSA amplification (Perkin-Elmer, NEL704 A, 1:50). Immunofluorescence slides were counterstained with DAPI, mounted with anti-fade Prolong Gold (Invitrogen) and imaged using an Olympus BX-51 fluorescence microscope.

### Cell lines and cultures

The GBM neurosphere lines HSR-GBM1, 040622 and 040821 were kind gifts from Dr. Angelo Vescovi and were propagated in serum-free media as previously described [59]. JHH-GBM10 and JHH-GBM14 neurosphere lines originated from tumors resected at Johns Hopkins University and were propagated in the same manner [60]. The pancreatic cancer line XPA3, a kind gift from Dr. Anirban Maitra (JHU), was propagated in RPMI with 10% FBS.

### Hypoxia time course analyses

HSR-GBM1, JHH-GBM10, JHH-GBM14, 040622 and 040821 neurosphere lines were plated at a density of 100,000 cells / ml media overnight, then exposed to 1% oxygen (hypoxia). Cells were harvested at 1, 6, 9, 22 and 48 h following hypoxic induction. Control cells were exposed to 20% oxygen (normoxia) and collected at the same time points. Approximately 4 × 10^6^ XPA3 cells were plated in 10-cm dishes, allowed to attach overnight, and exposed to hypoxia or normoxia for 48 h.

### Co-culture analyses

HSR-GBM1 and 040821 cells were transduced with lentiviruses encoding control pLenti6 vectors or JAG1 constructs. At least 5 × 10^5^ cells in each line (premix cells) were collected per line for RNA extraction. 5 × 10^6^ control vector cells were stained with the viable dye Carboxyfluorescein Diacetate Succinimidyl Ester (CFSE) according to manufacturer's instructions (Invitrogen, Carlsbad, CA) and co-cultured with the same number of JAG1 overexpressing cells in a T75 flask for 17 h. Co-cultured cells were then sorted into CFSE-positive (pLenti6) or CFSE-negative (plenti6-JAG1) populations by flow cytometry. Similarly, 6 × 10^6^ control vector and 6 × 10^6^ JAG1 XPA3 cells were co-cultured in a T75 flask for 17 h prior to sorting. The experiment was performed at least twice in each cell line.

### RNA analyses

RNA was extracted with Qiagen's RNeasy kit (Valencia, CA). RNA levels were assayed by real-time PCR analysis performed in triplicate with SYBR Green reagent (Applied Biosystems, Foster City, CA) according to the manufacturer's instructions on an I-Cycler IQ real-time detection system (Bio-Rad, Hercules, CA), with all reactions normalized to HPRT. To minimize contaminating genomic DNA, a 15-minute on-column DNase step (Qiagen RNase-free DNase kit) was included during RNA extraction. To measure the reaction efficiency, standard curves were generated for each primer pair using the standards of 0.05, 0.5, 5 and 50 ng of total starting RNA from HSR-GBM1 cells. In addition, all primer sets were verified by melting curves that demonstrated the amplification of a single product. Primer sequences are shown in Table 1.

**Table 1:** Primer sequences used for qPCR analyses

Primer Name	Sequence
HPRT Forward	5′- CTT TGC TGA CCT GCT GGA TT -3′
HPRT Reverse	5′- GTT GAG AGA TCA TCT CCA CC -3′
HES1 Forward	5′- GTG AAG CAC CTC CGG AAC -3′
HES1 Reverse	5′- CGT TCA TGC ACT CGC TGA -3′
HES5 Forward	5′- GTG CCT CCA CTA TGA TCC TTA AA -3′
HES5 Reverse	5′- AGT ACA AAG TCG TGC CCA CA -3′
HEY1 Forward	5′- TCT GAG CTG AGA AGG CTG GT -3′
HEY1 Reverse	5′- CGA AAT CCC AAA CTC CGA TA -3
HEY2 Forward	5′- AGA TGC TTC AGG CAA CAG GG -3′
HEY2 Reverse	5′- CAA GAG CGT GTG CGT CAA AG -3′
JAG1 Forward	5′- GAA TGG CAA CAA AAC TTG CAT -3
JAG1 Reverse	5′- AGC CTT GTC GGC AAA TAG C -3′
JAG2 Forward	5′- TGG GAC TGG GAC AAC GAT AC -3′
JAG2 Reverse	5′- ATG CGA CAC TCG CTC GAT -3′

### Protein analyses

Cells were first resuspended in TNE buffer containing 50 mM Tris, 150 mM NaCl, 5 mM EDTA, 1 mM PMSF, 10 mM NaF, 2 mM Na_3_VO_4_ and complete protease inhibitors (Roche). Resuspended cells were then lysed in an equal volume of TNE buffer containing detergents (1% NP-40, 1% deoxycholate, 2% SDS), and then sonicated for 20 seconds at 5-second intervals, and cooled on ice between intervals. Western blots containing at least 20 μg total protein per lane on a NuPAGE 4–12% BisTris gel (NP0321BOX) were electrophoresed in 1x NuPAGE MOPS SDS running buffer in an XCell SureLock mini gel apparatus (all from Invitrogen). Proteins were then transferred to Biorad's Immun-blot PVDF membrane in 1x NuPAGE transfer buffer in an X Cell II Blot module (Invitrogen). Membranes were blocked for 1 h at room temperature in 5% non-fat milk and incubated at 4°C overnight in primary antibodies containing 5% non-fat milk. JAG1 antibody (H114, sc-8303) was purchased from Santa Cruz Biotechnology and used at a 1:1000 concentration in blocking solution. V5 mouse monoclonal antibodies were purchased from Sigma. Mouse anti-rabbit GAPDH antibodies were purchased from Research Diagnostics (RDI-TRK5G4-6C5). Cleaved-NOTCH1 was purchased from Cell Signaling (4147) and used at a 1:800 concentration. β-actin was purchased from Santa Cruz Biotechnology and used at a 1:20,000 concentration in blocking solution. Secondary antibodies from KPL (peroxidase-conjugated goat anti-mouse [074–1806] or rabbit IgG [074–1506]) were diluted 1:5,000 in blocking solution. Blots were developed with enhanced chemiluminescence reagent (NEL103001EA, PerkinElmer).

### Plasmids and expression constructs

Constructs used for co-culture analyses and subsequent functional assays were cloned into the Gateway® pLenti6 plasmid containing a V5 epitope at the carboxy terminus. Full length human *JAG1* (M1 to V1218) in pLenti6 was constructed by transferring the *JAG1* gene from pENTR-JAG1 (Addgene) into the pLenti6 destination vector via an LR reaction according to manufacturer's instructions (Invitrogen). *JAG1ICD* (R1094 to V1218) was cloned into Gateway® entry vector via BP cloning. First, attB PCR products were generated using the primers in Table 2. PCR products were recombined into the Gateway® entry vector pDONR-221 in a BP reaction according to manufacturer's directions and then transferred to the pLenti6 destination vector via an LR reaction. Constructs were transformed into chemically competent Stbl3 bacteria for plasmid DNA amplification. Protein expression was verified by western blotting using anti-V5 antibodies. pLVU/RED [61] constructs were obtained by transferring the gene from a Gateway® entry/donor vector into the pLVU/RED destination vector via an LR reaction.

**Table 2:** Gateway primers used for designing attB PCR gene products Underlined sequence (ATG) denotes start codon.

Primer	attB1/B2 sequences	Gene specific sequences
**JAG1ICD_GTW_Forward**	GGGGACAAGTTTGTACAAAAAAGCAGGCTTCGA AGGAGATAGAACCATGGCG	CGGAAGCGGCGGAAGCCGGGCAGCC
**JAG1ICD_GTW_Reverse**	GGGGACCACTTTGTACAAGAAAGCTGGGTC	TACGATGTACTCCATTCGGTTTAAGC

### Lentivirus preparation and infection

Lentiviruses (from pLenti6, pLVU vectors) were produced in HEK 293T cells, followed by purification and concentration. Briefly, 4 plasmids (20 μg transfer vector with expression construct, 10 μg of packaging construct pMDL-g/pRRE, 5 μg pRSV-REV, 5 μg pMD2-VSVG) were used to co-transfect using 60 μl lipofectamine in 1.5 ml serum-free media, according to manufacturer's instructions in an 80% confluent T-75 flask. The next morning, the transfection media was replaced with fresh media containing 2% FBS in DMEM. Viruses were harvested at 48 h and 72 h and concentrated by PEG precipitation. Briefly, the viral supernatant was passed through a 0.45 μM low protein-binding filter. 50% PEG 8000 was added to the viral supernatant to a final concentration of 5%, and then 1.5 M NaCl was added to the virus-PEG mixture to a final concentration of 0.15 M and incubated overnight at 4°C. The precipitate was then centrifuged at 2000 g at 4°C for 30 min. Approximately 250,000 neurospheres were infected with concentrated virus overnight and changed to fresh media the next morning. After 3 days, transduced cells were selected with 2.5-5 μg/ml blasticidin antibiotics (pLenti6 constructs).

### Clonogenic assay

Clonogenic assays were performed in 6-well plates that were pre-coated with 500 μl of 12 mg/ml poly (2-hydroxyethyl methacrylate) (Sigma, St. Louis, MO) in 95% ethanol. The plates were allowed to dry at 37°C. Wells were first overlaid with 2 ml of 1.5% methyl cellulose (Fisher, M-352, Pittsburgh, PA) in NSA (MCNSA), and then incubated for 1 h at 37°C before plating. For each well, cells were resuspended with 1.5% MCNSA at a 1:3 ratio in a total volume of 2 ml. Cells were fed 0.5 ml of 1.5% MCNSA every 2 to 3 days. Subsequent sphere formation was monitored and scored by light microscopy after 10 to 13 days. Five random fields at 4x magnification were photographed in triplicate for each condition and all measurements were performed using a Motic AE31 light microscope equipped with a Moticam 2300 camera and analyzed using the Motic Images Plus 2.0 ML software (Motic Instruments, Richmond BC, Canada) as previously described [43].

## SUPPLEMENTARY FIGURE


