# Supplementary material for: Lateral inhibition of Notch signaling in neoplastic cells
Source: Oncotarget. 2014 Dec 9;6(3):1666–77. doi: 10.18632/oncotarget.2762 (PMC4359323; doi:10.18632/oncotarget.2762)
Supplement: Supplementary file 1 [file oncotarget-06-1666-s001.pdf]

SUPPLEMENTARY FIGURE

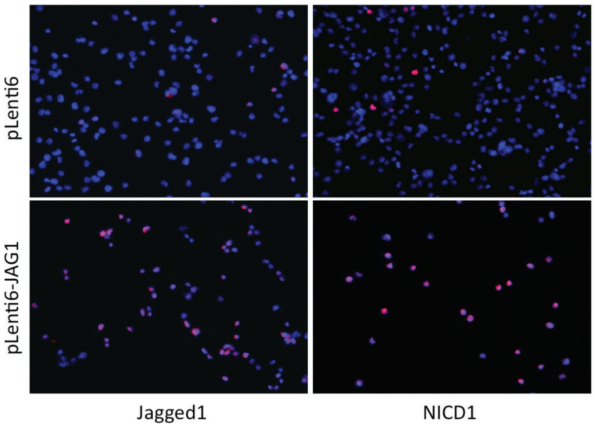

Supplemental Figure 1: JAG1 and NICD immunofluorescent expression in GBM1 pLenti6 and pLenti6-JAG1 expressing cells. Prepared as described in the Materials and Methods, these images are representative of 3 separate stainings. Individual antigen positive (red) and total cells (blue) were counted using the count tool in ImageJ software.
